# Grip Strength and the Risk of Cognitive Decline and Dementia: A Systematic Review and Meta-Analysis of Longitudinal Cohort Studies

**DOI:** 10.3389/fnagi.2021.625551

**Published:** 2021-02-04

**Authors:** Mengzhao Cui, Siwen Zhang, Yujia Liu, Xiaokun Gang, Guixia Wang

**Affiliations:** Department of Endocrinology and Metabolism, The First Hospital of Jilin University, Changchun, China

**Keywords:** grip strength, cognitive impairment, risk, meta-analysis, longitudinal studies

## Abstract

**Purpose:** Loss of grip strength and cognitive impairment are prevalent in the elderly, and they may share the pathogenesis in common. Several original studies have investigated the association between them, but the results remained controversial. In this systematic review and meta-analysis, we aimed to quantitatively determine the relationship between baseline grip strength and the risk of cognitive impairment and provide evidence for clinical work.

**Methods:** We performed a systematic review using PubMed, EMBASE, Cochrane, and Web of Science up to March 23, 2020, and focused on the association between baseline grip strength and onset of cognitive impairment. Next, we conducted a meta-analysis using a hazard ratio (HR) and 95% confidence interval (CI) as effect measures. Heterogeneity between the studies was examined using *I*^2^ and *p-*value. Sensitivity analyses and subgroup analyses were also performed, and publication bias was assessed by Begg's and Egger's tests.

**Results:** Fifteen studies were included in this systematic review. After sensitivity analyses, poorer grip strength was associated with more risk of cognitive decline and dementia (HR = 1.99, 95%CI: 1.71–2.32; HR = 1.54, 95%CI: 1.32–1.79, respectively). Furthermore, subgroup analysis indicated that people with poorer strength had more risk of Alzheimer's disease (AD) and non-AD dementia (HR = 1.41, 95%CI: 1.09–1.81; HR = 1.45, 95%CI: 1.10–1.91, respectively).

**Conclusions:** Lower grip strength is associated with more risk of onset of cognitive decline and dementia despite of subtype of dementia. We should be alert for the individuals with poor grip strength and identify cognitive dysfunction early.

## Introduction

Physical performance gradually declines with aging, and loss of grip strength is also a well-recognized manifestation of age-related motor decline and of geriatric syndromes such as sarcopenia and frailty (Buchman et al., 2007). Measure of grip strength is noninvasive and widely available (Ferlay et al., 2019), therefore grip strength measurement is often applied to reflect upper muscle strength in clinical setting. Being sensitive to age-related changes and changes in biological function, grip strength is not only an indicator of muscle strength but also of biological vitality (MacDonald et al., 2004). Furthermore, cognitive decline and dementia are common outcomes of aging that significantly affect the quality of life of the elderly people. Cognitive decline is frequently regarded as a prodrome to dementia, but exists on a continuum with normal aging (Heward et al., 2018). Individuals with cognitive decline are nevertheless at high risk of developing cognitive impairment and dementia.

It is reported that sarcopenia leads to physical inactivity, and physical decline has been consistently associated with future cognitive decline (Cabett Cipolli et al., 2019). Inflammatory and hormonal pathway and brain atrophy might be the explanation for the association between sarcopenia and cognitive impairment (Iannuzzi-Sucich et al., 2002; Schaap et al., 2006). Besides, frailty is common in the elderly. Frailty was defined as a clinical syndrome in which three or more of the following criteria were present: unintentional weight loss (10 lbs in past year), self-reported exhaustion, weakness (low grip strength), slow walking speed, and low physical activity (Fried et al., 2001). Frailty and AD may share underlying pathogeneses, and the rates of change of frailty and cognition over time are strongly associated with the same brain pathologies, such as the presence of macroinfarcts, Alzheimer's disease pathology, and nigral neuronal loss (Buchman et al., 2014). Besides, sarcopenia, frailty and cognitive impairment are all correlated to aging, oxidative stress and inflammation and so on. A recent meta-analysis (Peng et al., 2019) indicated that sarcopenia was associated with an increased risk of cognitive impairment independent of study population, the definition of sarcopenia and cognitive impairment, but only cross-sectional studies were included in this analysis, and the association between three components of sarcopenia and cognitive function was not identified. Another systematic review (Zammit et al., 2019) showed that cognitive function and grip strength (as the measure of frailty) declined with aging, but with little to no evidence for longitudinal associations between rates of change of both.

Older individuals with low handgrip strength often show symptoms that can interfere with cognitive and physical performance, such as declines in mobility and balance and impairment in executive function and memory (Firth et al., 2018). Grip strength, as an important component of sarcopenia and frailty, was also reported to be associated with cognitive aging (Zammit et al., 2019), its relation to cognitive impairment is of great interest, therefore, we focused on the longitudinal relationship between baseline grip strength and the risk of cognitive impairment. It's of great benefit that regarding grip strength as an observational indicator, and discerning cognitive impairment at the early stages. Several studies have reported that poorer grip strength was associated with cognitive decline and onset of dementia (Heward et al., 2018; Jeong and Kim, 2018; Jeong et al., 2018), while some studies found no relationship between grip strength and cognitive dysfunction (Sibbett et al., 2018; Doi et al., 2019). This meta-analysis aimed to investigate the relationship between grip strength and onset of cognitive impairment including cognitive decline and dementia, in order to identify dementia earlier especially in persons who have lower grip strength and promote more appropriate planning for prevention and treatment for dementia.

## Methods

### Selection of Studies

Comprehensive literature searches in PubMed, EMBASE, Cochrane and Web of Science from their inception to March 23, 2020, were performed using a combination of Medical Subject Heading (MeSH) terms with related free text terms (“grip strength” OR “hand strength” OR “muscle strength”) AND (“cognitive impairment” OR “cognitive dysfunction” OR “cognitive decline” OR “cognitive disorder” OR “dementia”). Specific search strategies for each database can be found in Supplementary Material 1. Only those studies that met the following inclusion criteria were selected: (1) it was an original longitudinal cohort study that aimed at the association between grip strength and cognitive impairment; (2) included validated and recognized tests that assessed cognition and grip strength; (3) original research articles developed with humans and in English; (4) grip strength as the exposure; (5) cognitive impairment as the outcome of observation; (6) HR and 95% CI were available. Review articles, case reports, animal studies, abstracts and chapters or books were not included. The studies searched in different databases were combined, and duplicate references were removed. If there was a duplication or overlap of the study group, then the study with the longest follow-up or had better quality was selected for the analysis. After removing duplicate papers, two authors (MC and SZ) independently screened the titles and abstracts of the identified searches and followed by a full-text review of potentially eligible articles. Furthermore, disagreements in study selection were judged and resolved by consensus among the involved authors. The current systematic review and meta-analysis followed the meta-analysis of observational studies in epidemiology (MOOSE) guidelines (Stroup et al., 2000).

### Data Extraction

The first author's name, year of publication, study design, population of study, sample size, follow-up period, follow-up rate, assessment of muscle strength, evaluation of cognitive function, outcome, statistical model, the adjusted variables included in the regression model and HRs ad 95%CI were extracted from each reference.

### Quality Assessment

Since all of the included studies are observational cohort study design, the adapted version of the Newcastle-Ottawa Scale (NOS) for quality assessment with 8-items for cohort studies was applied (Stang, 2010). A score of six or above was considered as high quality. If the two authors were unable to reach a consensus on the quality of studies, the corresponding author was consulted.

### Statistical Analysis

Pooled mean effect size was calculated using meta-analysis software Stata 15 SE. First, determine whether there is heterogeneity among the original studies. Study heterogeneity was measured using the Cochran's Q and I-squared statistics, assuming that a *p* < 0.10 for the former and a value ≥ 50% for the latter indicated a significant and substantial heterogeneity. Second, due to the heterogeneity, the random-effect model was used to calculate pooled effect size of HRs and 95%CI. Furthermore, sensitivity analysis was performed to evaluate the robustness of results, in which pooled estimates were calculated after removing one study in each turn. Besides, subgroup analyses by type of cognitive impairment, cognition assessment, age, gender, population source were also performed to exclude effect of heterogeneity. Finally, publication bias was assessed using Begg's and Egger's tests.

## Results

### Literature Search

**Figure 1:**
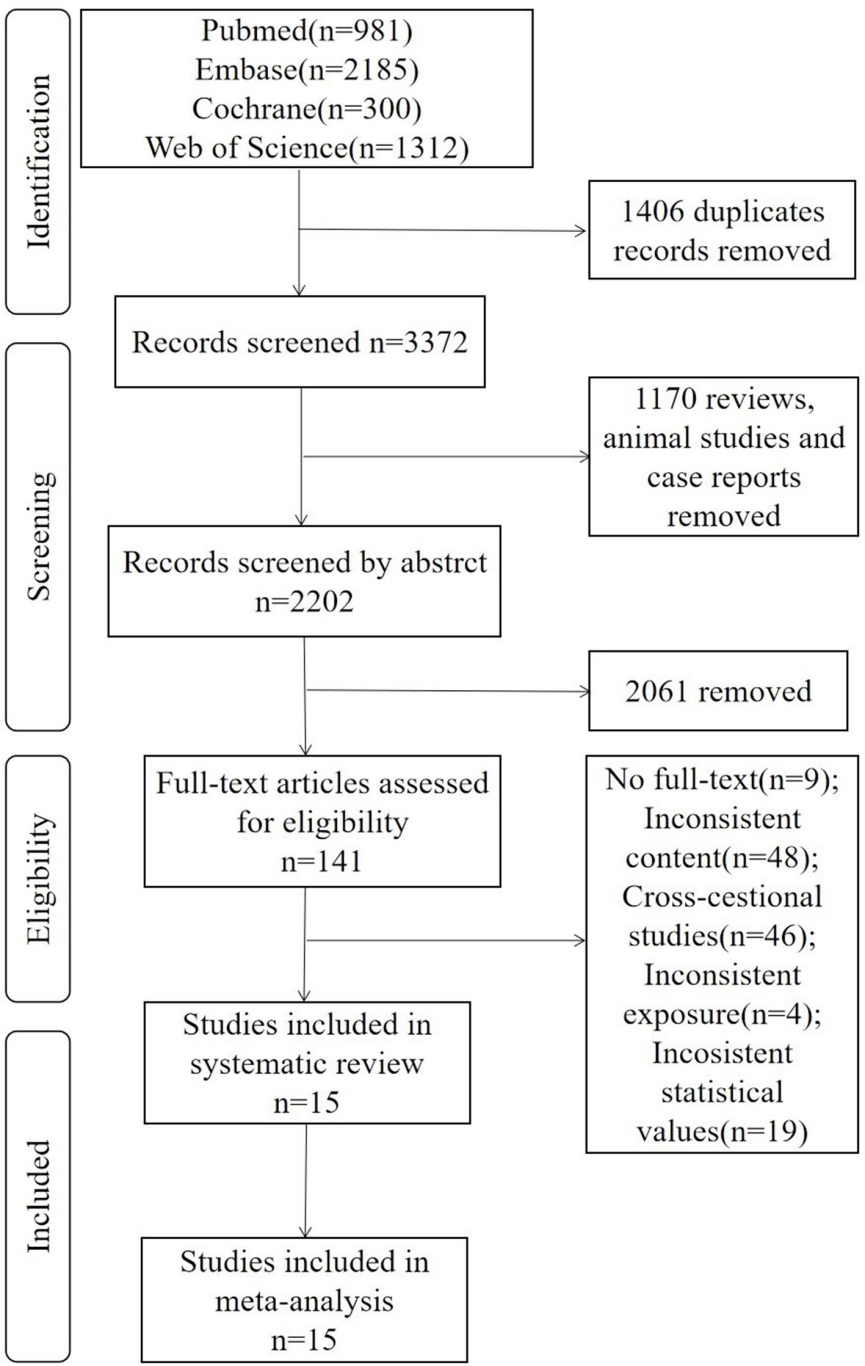
Flow diagram of the literature search.

A total of 4,778 articles were retrieved from Pubmed (981), Embase (2,185), Cochrane (300), and Web of Science (1,312). Among all the articles, 1,406 duplicates, 1,170 reviews and comments, books, animal studies and case reports were removed. After reading abstracts, 2,061 articles were excluded. Finally, after full-text reading, 15 articles (Buchman et al., 2007; Boyle et al., 2009; Sattler et al., 2011; Gray et al., 2013; Camargo et al., 2016; Moon et al., 2016; Veronese et al., 2016; Hooghiemstra et al., 2017; Heward et al., 2018; Jeong and Kim, 2018; Jeong et al., 2018; Sibbett et al., 2018; Doi et al., 2019; Kim et al., 2019; Hatabe et al., 2020) were included in this systematic review and meta-analysis (Figure 1).

### Characteristics of the Studies and Participants

Characteristics of all the studies included are listed in Table 1. The 15 included studies had 27,588 individuals, with the age of most studies more than 55 years. The sample size ranged from 263 to 6,435. Six studies were conducted in Asia, four studies were conducted in Europe, four were conducted in North America and one in Africa. The subjects of the studies were from the community-dwelling setting (13 studies), religious communities (one study), and memory clinic (one study). Ten articles regarded dementia (including AD, Vascular Dementia, non-AD dementia) as outcome variable and five were cognitive decline based on the decline of cognitive assessment scales. As for the diagnosis of dementia, two studies were based on medical record, four studies based on Diagnostic and Statistical Manual of Mental Disorders (Fourth Edition) (DSM-IV), and National Institute of Neurological Diseases and Stroke and the Alzheimer's Disease and Related Disorders Association (NINCDS-ADRDA) criteria for AD. Besides, for cognitive assessment, cognitive scales were used in the most of the studies, such as mini-mental state examination (MMSE). As to the cognitive status at baseline, participants were with subjective or objective cognitive impairment but no dementia (one study), without dementia (eight studies) and the residuals were without cognitive dysfunction. Most of studies conducted multivariable adjusted analyses with important confounders, including age, gender, education, and comorbidities. All studies were longitudinal cohort study design.

**Table 1:** Characteristics of the included studies.

**Study**	**Country, setting**	**Population, sample size**	**Follow-up period(year)**	**Muscle strength assessment**	**Cognitive function assessment**	**Outcome**	**Statistical model**	**Effect measures**
Doi et al. (2019)	Japan, community-dwelling	Older adults ≥ 65 years,52% women without dementia,4,086	3.6	Measured once; Smedley-type handheld dynamometer	Recorded data collected by the Japanese Health Insurance System	Incident dementia	Cox proportional hazards models	HR 1.39 (0.89–2.17)
Jeong et al. (2018)	Korea, community-dwelling	Older women without cognitive impairment aged 65 years or older, 544	8	Measured twice for each hand and averaged the maximum value from each hand; dynamometer	MMSE of Korean version scores; cognitive dysfunction: MMSE <2 4	Cognitive decline	Logistic and linear regression analyses	aOR 2.28 (1.23–4.24)
Sibbett et al. (2018)	Scotland, community-dwelling	Aged ~79 years (57% women), without dementia, 416	16	Measured three times in the dominant hand and used the best; Jamar Hydraulic Hand Dynamometer	Collected from death certificates, electronic hospital records, and clinical reviews	Incident dementia	Cox Regression	HR 0.98 (0.94–1.02)
Heward et al. (2018)	Tanzania, community-dwelling	Adults aged 65 years and over without cognitive dysfunction (57% women), 305	2	Measured three times in the dominant hand and used the best; Jamar hydraulic hand dynamometer	IDEA cognitive screen; A score of ≤7: probable cognitive impairment; A score of 8–9: possible cognitive impairment	Cognitive decline	Multivariable binary logistic regression	OR 3.93 (1.70–9.11)
Jeong et al. (2018)	Korea, community-dwelling	Adults aged ≥ 45 years without cognitive dysfunction (50% women),6435	5.21	The mean of the maximum handgrip strength from both hands	MMSE of Korean version score; cognitive dysfunction: MMSE <24	Incident cognitive dysfunction	Cox proportional hazard models	HR 1.39 (1.17–1.67)
Hooghiemstra et al. (2017)	Netherlands, memory clinic	Age of 55 years or older and subjective or objective cognitive impairment but no dementia (35% women), 263	2.1 ± 1.2	Measured twice for the dominant hand and used the mean value; hydraulic hand dynamometer	MMSE; RAVLT; VAT; Digit Span; LDST; SCWT; TMT; MCI: based on clinical judgment, the Petersen criteria	Progression to MCI or dementia for SCD and to dementia for MCI	Cox proportional hazard models	HR 1.05 (0.76–1.46); Stratified by baseline cognitive function: SCD, HR 1.13 (0.63–2.03); MCI, HR 1.06 (0.70–1.60)
					Dementia: DSM-IV-TR; AD: NIA-AA criteria			Stratified by age: ≤65y: HR 1.05 (0.58-1.87);>65y: HR 1.09 (0.75–1.60)
Camargo et al. (2016)	USA, community-dwelling	Framingham Offspring, mean age 62 ± 9 years (54% women), 2,046	6.5	Measured three times of each hand and recorded the maximum; Jamar Hydraulic Hand Dynamometer	MMSE and standardized neuropsychological test battery; Dementia: DSM-IV, and along with symptoms for at least 6 months	Incident dementia and AD	Cox proportional hazard models	For the entire study sample: Dementia: HR 2.17 (1.00–4.69); AD: HR 2.75 (1.18–6.39)
					AD, NINCDS-ADRDA			≥65 years: Dementia: HR 2.38 (1.05–5.39); AD:HR 3.22 (1.31–7.90)
Veronese et al. (2016)	Italy,community-dwelling	Older adults aged ≥65 years (59% women), 1,249	4	Measured three times of each hand and used the maximum; JAMAR hand-held dynamometer	30-items MMSE	Onset of cognitive decline	Multinomial logistic regression	AD: HR 3.22 (1.31–7.90)
Gray et al. (2013)	USA, community-dwelling	Aged 65 years and older without dementia (60% women), 2,619	6.5 ± 3.9	Measured three times in the dominant hand and used the best; handheld dynamometer	CASI; a cutoff value of 86 on the CASI to identify individuals for dementia evaluation.	Incident dementia, AD and non-AD dementia	Cox proportional hazards regression models	Dementia: HR 1.06 (0.87–1.29); Possible or Probable AD: HR 1.04 (0.84–1.28); Non-AD Dementia: HR 1.28 (0.77–2.11)
Sattler et al. (2011)	Germany, community-dwelling	Healthy participants from the 1930–32 birth cohort, an average age of 74 years, 323	12	Assessed with the aid of the “Martin-Vigorimeter”, subjects press a ball alternating between two hands for four trials and used the maximum.	MCI: AACD criteria; AD: the NINCDS-ADRDA criteria; VaD: the NINDS-AIREN criteria	Prevalence of MCI/AD	Logistic regression analyses	OR 1.00 (0.99–1.01)
Boyle et al. (2009)	USA, community-dwelling	older persons without dementia, mean age 80.3 ± 7.5 years (75% women), 970	3.6	Measured bilaterally and used the average using the Jamar® hydraulic hand dynamometers.	21 tests and MMSE; AD: NINCDS-ADRDA criteria; MCI: had cognitive impairment but did not meet criteria for dementia	Risk of AD	Core proportional hazards model	HR 0.61 (0.47–0.80)
Buchman et al. (2007)	USA, religious communities	Older Catholic clergy members (70% women), 843, excluding 34 participants who had a clinical stroke at baseline or during the course of the study; ≥67 years	5.7	Measured twice for each hand and averaged the four trials. Jamar hydraulic hand dynamometer.	20 cognitive performance tests. Dementia: NINCDS-ADRDA criteria; AD: a history of cognitive decline and evidence of impairment in two or more domains of cognition, and one must be memory.	Risk of AD	Cox proportional hazards models	HR 0.981 (0.968–0.994)
Kim et al. (2019)	Korea, community-dwelling	Aged 50 and over (54.5% women), 5,995	8	Measured twice for each hand and used the mean value. Handheld dynamometer.	Korean version of the MMSE; Mild cognitive impairment: K-MMSE score of 18–23; Dementia: K-MMSE score ≤ 17	Cognitive impairment	2-years lagged general estimating equations (GEE)	OR 0.499 (0.422–0.589)
Moon et al. (2016)	Korea, community-dwelling	Aged 65 years or more, cognitively normal (47% women), 297	5	Measured twice consecutively for the dominant hand with a 1-min interval, and used the averaged value.	CERAD-K Clinical Assessment Batter and M.I.N.I.; MCI: the diagnostic criteria proposed by the International Working Group. Dementia: DSM-IV	Progression of cognitively normal to MCI or dementia	Multivariate binary logistic regression analysis	HR 1.337 (0.281–6.368)
Hatabe et al. (2020)	Japan, community-dwelling	Community-dwellers of late-life aged 60–79 without dementia (57% women),1055	14.6 ± 7.3	Measured twice for each hand and used the maximum value; Smedley hand dynamometer	MMSE and Hasegawa's Dementia Scale revised; Dementia: DSM-IV; AD: NINCDS-ADRDA; VaD: NINDS-AIREN criteria	Risk of developing dementia	Cox proportional hazards models	Total dementia: HR1.66 (1.29-2.13); AD: HR 1.94 (1.41-2.67); VaD: HR 2.07 (1.32–3.25)
		Community-dwellers of middle-life aged 45–64 without dementia, 835						Total dementia: HR 1.29 (0.996–1.67);AD:HR 1.46 (1.05–2.03); VaD: HR 1.07 (0.66–1.74)

*MMSE, mini-mental state examination; IDEA, Identification and Intervention for Dementia in Elderly Africans; RAVLT, Rey Auditory Verbal Learning Test; VAT, Visual Association Test; LDST, Letter Digit Substitution Test; SCWT, Stroop Color Word Test; TMT, Trail Making Test; DSM-IV-TR, Diagnostic and Statistical Manual of Mental Disorders, fourth Edition, text revision; NIA-AA, National Institute of Aging-Alzheimer's Association; CASI, Cognitive Abilities Screening Instrument; AACD, aging-associated cognitive decline; NINCDS-ADRDA, National Institute of Neurological Diseases and Stroke and the Alzheimer's Disease and Related Disorders Association; VaD, Vascular Dementia; NINDS-AIREN, National Institute of Neurological Diseases and Stroke and Association Internationale pour la Recherche et l'Enseignement en Neurosciences; DSM-IV, Diagnostic and Statistical Manual of Mental Disorders, fourth Edition; CERAD-K, Korean Version of the Consortium to Establish a Registry for Alzheimer's Disease; M.I.N.I., The Mini-International Neuropsychiatric Interview*.

### Quality Assessment

According to NOS scale, all of the included studies were of high quality (six studies for nine stars, five studies for eight stars and four studies for seven stars). Details were presented in Supplementary Table 1.

### Meta-Analysis Findings

#### Association Between Grip Strength and Risk of Cognitive Decline

**Figure 2:**
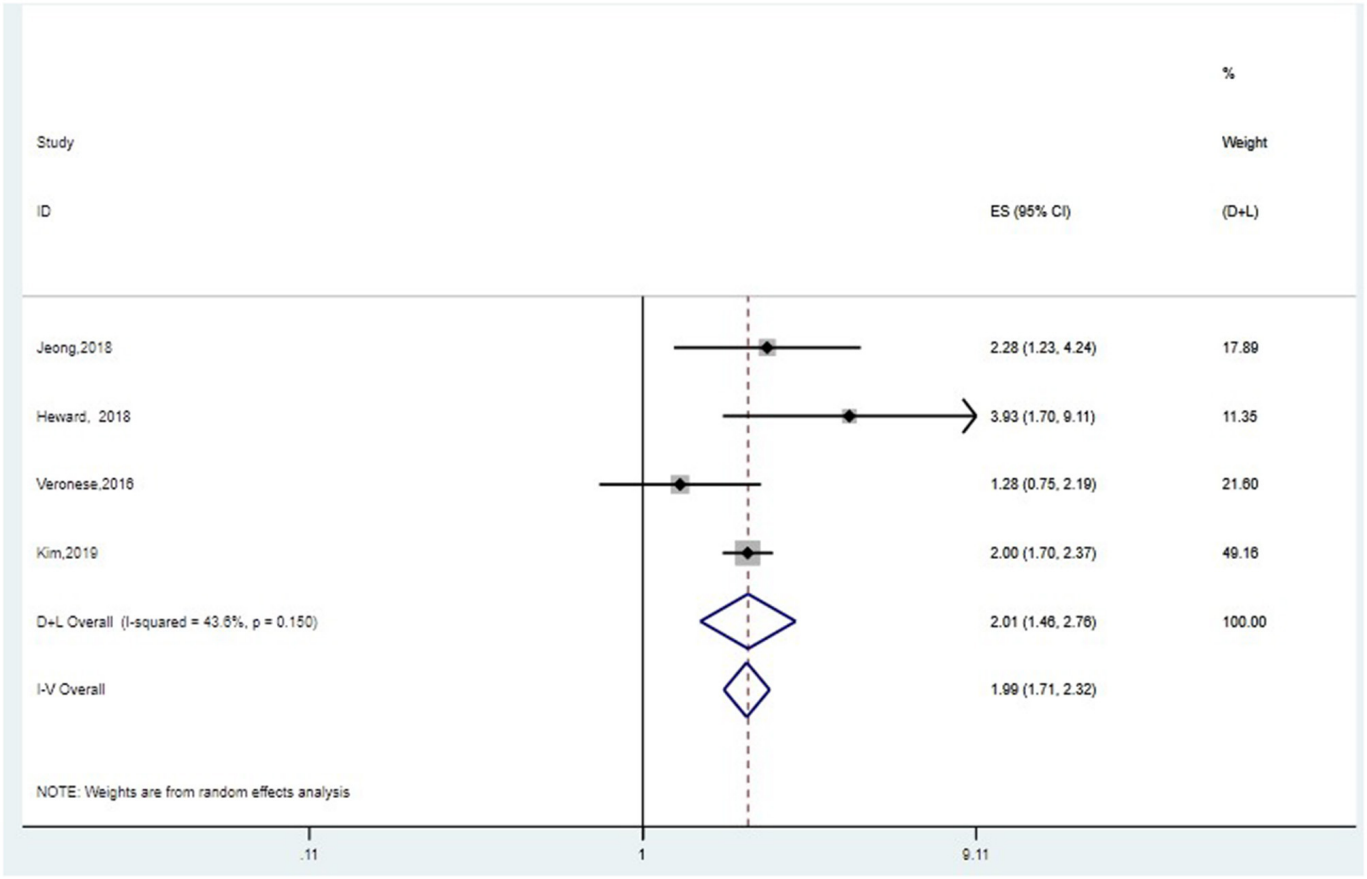
Meta-analysis of the association between grip strength and cognitive decline after removing one study. The pooled HRs of four studies on the association between grip strength and cognitive decline was 1.99, *95%CI*: 1.71–2.32 (*I*^2^ = 43.6%, *P* = 0.150) with fixed-effect model.

Five studies assessed the association between grip strength and cognitive decline. Poorer grip strength was associated with higher risk of cognitive decline (HR = 1.80, *95%CI:* 1.35–2.40, *I*^2^ = 72.3%, *P* = 0.006) (Supplementary Figure 1A). Sensitivity analysis indicated that effects of Jeong and Kim (2018) might be the source of heterogeneity (Supplementary Figure 1B). On one hand, the population of this study was relatively young, so the onset of cognitive decline might not be very obvious. On the other hand, cox proportional hazard models were used in this study, which were different from other included studies. After removing this study, the pooled effect size of HRs was 1.99, *95%CI:* 1.71–2.32 (*I*^2^ = 43.6%, *P* = 0.150) with fixed-effect model (Figure 2). Begg's and Egger's tests produced *P-*values of 0.308 and 0.826, respectively, suggesting the absence of publication bias (Supplementary Figure 1C).

#### Association Between Grip Strength and Risk of Dementia

**Figure 3:**
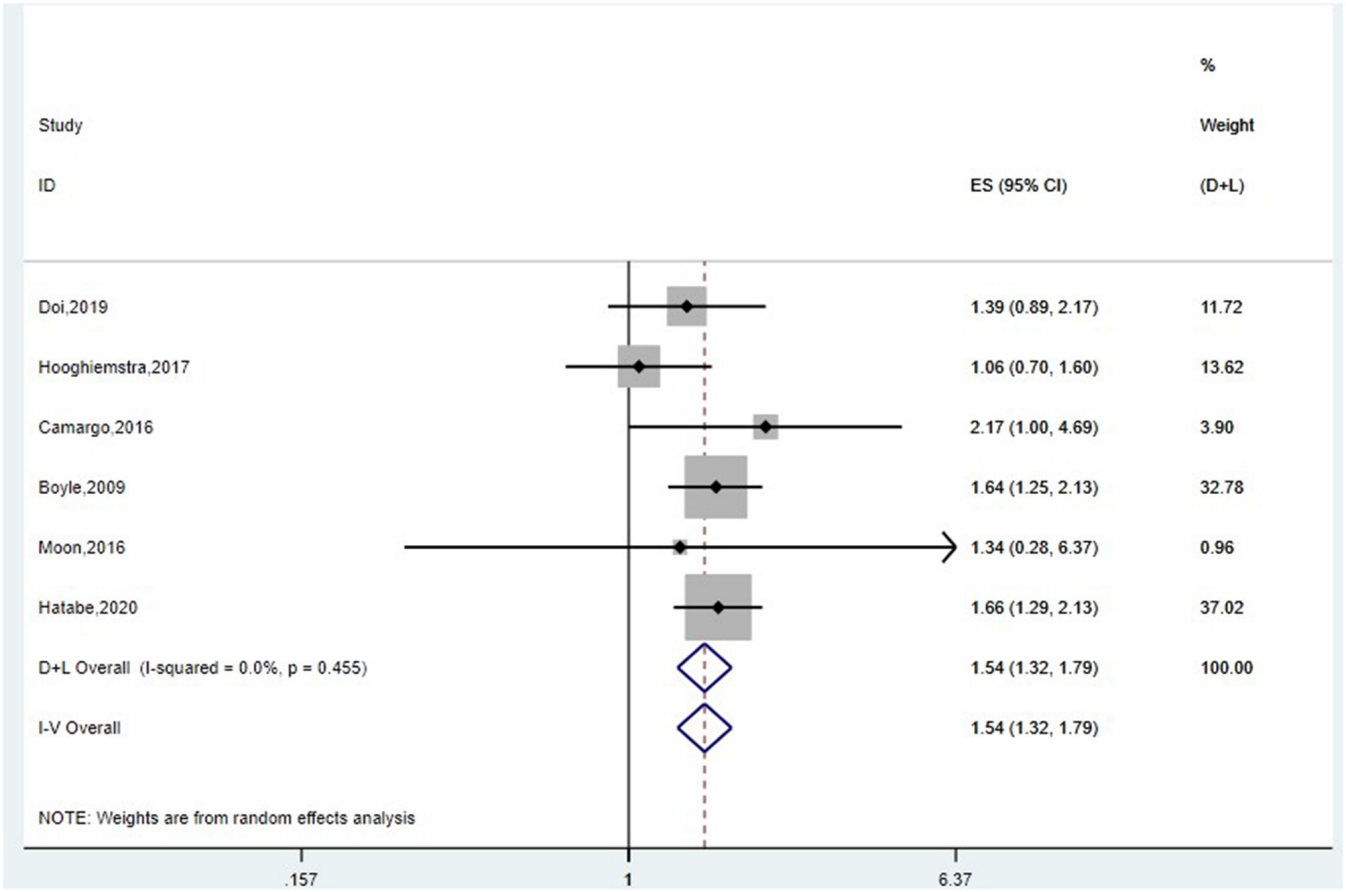
Meta-analysis of the association between grip strength and dementia after removing four studies. The pooled HR of six studies on the association between grip strength and dementia was 1.54 (95%CI: 1.32–1.79, *I*^2^ = 0.0%, *P* = 0.455).

First, we conducted meta-analysis for 10 studies, of which outcome was dementia. If the studies included several outcomes, we selected the HRs of dementia, not AD or VaD. Furthermore, we chose the HRs of the entire samples instead of HRs of stratification by age or gender. The pooled HR of random-effect model was 1.03 (*95%CI:* 0.99–1.07, *I*^2^ = 78.1%, *P* = 0.000) (Supplementary Figure 2A). After sensitivity analysis (Supplementary Figures 2B–F), four articles (Buchman et al., 2007; Sattler et al., 2011; Gray et al., 2013; Sibbett et al., 2018) were removed, and the final pooled HR of fixed-effect model was 1.54 (*95%CI:* 1.32–1.79, *I*^2^ = 0.0%, *P* = 0.455) (Figure 3). People with poorer grip strength had more risk for suffering dementia. Begg's and Egger's tests produced *P*-values of 1.0 and 0.744, respectively, suggesting the absence of publication bias (Supplementary Figure 2G).

Next, if the studies include several outcomes, then we extracted the HRs of each subtype of dementia, that is dementia, AD or non-AD dementia. The pooled HR of random-effect model was 1.08 (*95%CI:* 1.03–1.13, *I*^2^ = 78.2%, *P* = 0.000) (Supplementary Figure 3).

Based on the above results, we performed subgroup analysis by subtype of dementia, study region, sample size, setting and grip strength assessment (Table 2). People with poorer strength had more risk of AD and non-AD dementia (HR = 1.41, *95%CI:* 1.09–1.81; HR = 1.45, *95%CI:* 1.10–1.91, respectively). Studies from Asia had positive results (HR = 1.59, *95%CI:* 1.28–1.97), but results from European and North American studies were negative. Studies of sample size ≥1,000 came to a conclusion that declined grip strength was associated with high prevalence of dementia (HR = 1.41, *95%CI:* 1.04–1.91). Grip strength assessment might also influence the outcome. After subgroup analysis, heterogeneity among studies still remained, while we conducted Begg's and Egger's tests of each subgroup, suggesting that our results had almost no publication bias (Supplementary Figures 4A–J). However, we still need expand the literature numbers to make further analysis.

**Table 2:** Subgroup analyses based on subtype of dementia, study region, sample size, setting and grip strength assessment.

**Variables**	**Number of studies**	**Meta-analysis (HR, 95%CI)**	**Heterogeneity**	
			***I*^**2**^**	***P***
**Subtype of dementia**				
AD	6	1.41 (1.09–1.81)	86.7%	0.000
non-AD dementia	3	1.45 (1.10–1.91)	51.7%	0.126
**Study region**				
Asia	3	1.59 (1.28–1.97)	0.0%	0.775
Europe	3	1.00 (0.99–1.01)	0.0%	0.617
North America	4	1.23 (0.97–1.57)	81.3%	0.001
**Sample size**				
≥1,000	4	1.41 (1.04–1.91)	68.6%	0.023
<1,000	6	1.01 (0.98–1.04)	74.6%	0.001
**Setting**				
Community	8	1.12 (1.02–1.22)	80.7%	0.000
Clinic	1	-	-	-
Both community and clinic	1	-	-	-
**Grip strength assessment**				
Dominant hand with handheld dynamometer	5	0.99 (0.95–1.03)	0.0%	0.533
Both hands with handheld dynamometer	4	1.47 (1.01–2.13)	90.1%	0.000
Both hands with a ball	1	1.00 (0.99–1.01)	-	-

## Discussion

This meta-analysis focused on the longitudinal association between grip strength and risk of cognitive impairment including two aspects, cognitive decline and dementia. Poorer grip strength was associated with higher risk of cognitive decline (HR = 1.99, *95%CI:* 1.71–2.32, *I*^2^ = 43.6%, *P* = 0.150) and with onset of dementia (HR = 1.54, *95%CI:* 1.32–1.79, *I*^2^ = 0.0%, *P* = 0.455). Furthermore, subgroup analysis indicated that people with poorer strength had more risk of AD and non-AD dementia (HR = 1.41, *95%CI:* 1.09–1.81; HR = 1.45, *95%CI:* 1.10–1.91, respectively). This meta-analysis quantitatively accumulated evidence from longitudinal studies suggesting that grip strength was inversely associated with the risk of cognitive decline and dementia in the middle-aged and elderly populations. In other words, grip strength may be an early indirect non-cognitive marker of subsequent cognitive decline or onset of dementia. Higher grip strength may reflect the integrity of neuromuscular system and higher resistance to oxidative stress and inflammation may extend to preservation of cognitive function (Weaver et al., 2002). Therefore, we could take measurement of grip strength as the initial screening, and for people who are identified as having poor grip strength, we should remain alert to the possibility of their cognitive problems.

This is the first study to quantitatively determine the association between grip strength and risk of cognitive impairment. Meta-analyses suggested a positive relationship between sarcopenia and cognitive impairment (Cabett Cipolli et al., 2019; Peng et al., 2019), but which component of sarcopenia played a dominant role was not elucidated. However, lower muscle mass was thought not to be associated with high risk of cognitive dysfunction. A cross-sectional study also showed that lower-extremity functioning, rather than skeletal muscle mass, is closely related to multiple cognitive domains (Ishii et al., 2019). In the Epidemiologie de l'Osteoporose cohort, decreased muscle mass alone was not associated with cognitive dysfunction after seven years of follow-up (van Kan et al., 2013). In another community-based prospective cohort study, low muscle mass did not increase the risk of dementia in older adults during the 5 years of follow-up (Moon et al., 2016). Furthermore, a meta-analysis (Quan et al., 2017) suggested slow or decreased walking pace is significantly associated with elevated risk of cognitive decline and dementia in elderly populations. Based on these findings, lower grip strength and gait speed may be underlying factors for explaining the relationship between sarcopenia and cognitive impairment. Besides, except for the studies included in this meta-analysis, there are also other studies which are consistent with our outcome. Chou et al. reported that a low handgrip strength could predict 10-years cognitive decline among community-dwelling older people (Chou et al., 2019). Handgrip strength predicted accelerated 1-year decline in cognitive function, assessed by Clock Drawing Test Performance, in a sample of older non-demented adults (Viscogliosi et al., 2017). Weaker grip strength in older women was associated with cognitive decline over a 4-years period (Auyeung et al., 2011). Importantly, more studies should be included in meta-analysis in the future.

In current study, as for cognitive decline, an article (Jeong and Kim, 2018) was removed after sensitivity analysis, due to the relatively younger population, and cox proportional hazard models used in this study, which were different from other included studies. In this way, the fixed-effect model was conducted and suggested poorer strength was significantly associated with cognitive decline. Furthermore, in the aspect of onset of dementia, first we regarded entire HRs of dementia as the outcome, after sensitivity analysis, four articles were removed. Sattler et al. (2011) assessed grip strength with the aid of the “Martin-Vigorimeter”, and participants pressed a ball alternating between two hands, which was different from other assessment with hydraulic hand dynamometer. Population of Buchman et al. (2007) was from older Catholic clergy members, whose physical fitness and cognitive function might be different from the community-dwelling. Sibbett et al. (2018) indicated that due to the retrospective dementia cases from records, the date of onset of dementia might not be accurate. Besides, the missed cases might also affect the outcome. Finally, diagnosis of dementia of Gray et al. (2013) was based on Cognitive Abilities Screening Instrument (CASI), while others were based on diagnostic criteria of dementia and AD. Therefore, these four articles might be the source of heterogeneity. And the final pooled HR was 1.54, *95%CI:* 1.32–1.79, *I*^2^ = 0.0%, *P* = 0.455. Next, since some of the studies included several outcomes of dementia, we extracted HRs of each outcome respectively and conducted meta-analysis. The pooled HR of random-effect model was 1.08 (*95%CI:* 1.03–1.13, *I*^2^ = 78.2%, *P* = 0.000). Based on the results, we performed subgroup analysis to find out the source of heterogeneity. People with poorer strength had more risk of AD and non-AD dementia (*HR* = *1.41, 95%CI: 1.09–1.81; HR* = *1.45, 95%CI: 1.10–1.91*, respectively), which was consistent with previous entire outcome. Studies from Asia and sample size ≥1,000 had positive results (*HR* = *1.59, 95%CI: 1.28–1.97; HR* = *1.41, 95%CI: 1.04–1.91*, respectively) and grip strength assessment might also influence the outcome (both hands with handheld dynamometer, *HR* = *1.47, 95%CI: 1.01–2.13*). Subgroup analysis could not eliminate the heterogeneity, while we conducted Begg's and Egger's tests of each subgroup, suggesting that our results had almost no publication bias. We still need expand our literature numbers to reduce the heterogeneity.

As to the mechanisms of this finding, several potential aspects may account for the relationship between grip strength and cognitive impairment. First, oxidative stress and inflammation are well-known to be directly related to cognitive decline (Glade, 2010), and the onset of cognitive dysfunction (Pedersen and Febbraio, 2012). Accumulating evidences have shown that skeletal muscle has a secretory role of cytokines and other peptides, such as brain-derived neurotrophic factor (BDNF), interleukin-6 (IL-6), IL-8, IL-15, et al., which are involved in inflammatory processes (Pedersen and Febbraio, 2012) and loss of muscle strength (Aleman et al., 2011). Decline in muscle mass and strength may also reduce expression of BDNF, insulin-like growth factor-1, both of which are considered to play a role in learning and neural plasticity (Pedersen and Febbraio, 2012), so lower grip strength may cause cognitive impairment. Furthermore, weak handgrip strength may be an early sign of cognitive impairment, as handgrip strength could be reflected by change of nervous system activity or white matter integrity (Jeong et al., 2018). Previous findings suggest that brain structure may be associated with grip strength and cognitive changes (Sachdev et al., 2005, 2009). Besides, vitamin D (Vit D) may play a role between muscle and cognitive function. Vit D deficiency is strongly linked to muscle weakness and loss, suggesting that hypovitaminosis D in the elderly may be an important factor in the development of sarcopenia (Berchtold et al., 2000; Lappe and Binkley, 2015; Verde et al., 2019). It is found that the Vit D receptor is also expressed in the central nervous system (CNS) and that CNS is able to synthetize calcitriol *per se* due to the expression of the 25-hydroxylase and 1α-hydroxylase, which has raised the hypothesis that Vit D may have a role in brain health and in cognitive performance (D'amelio and Quacquarelli, 2020).

There are some limitations in our study. First, evidence gathering from observational longitudinal studies cannot account for the cause and effect between grip strength and cognitive dysfunction. This meta-analysis aimed at the association between baseline grip strength and onset of cognitive impairment compared with cognitive performance of baseline. It seems that lower grip strength may cause cognitive decline and dementia, but it's still not enough to determine causality. Some suggested that cognitive function might precede muscle weakness (Jeong et al., 2018), others indicated that strength capacity and cognitive function might parallel each other (McGrath et al., 2020). However, based on the negative association between grip strength and the risk of cognitive impairment, it's significative for clinical work that taking grip strength as a tool to identify cognitive dysfunction early. Second, heterogeneity among studies could not be absolutely eliminated after subgroup analysis, while we performed sensitivity analysis and bias test, all of which suggested our results were reliable. However, we should still focus on the articles in this field to expand our literature sources.

In conclusion, poorer grip strength is associated with more onset of cognitive decline and dementia even subtype of dementia. Early prevention of cognitive impairment may focus on the better recognition and improvement of grip strength. More prospective studies need to be conducted to clarify the cause and effect between grip strength and cognitive impairment.

## Data Availability Statement

The original contributions presented in the study are included in the article/Supplementary Material, further inquiries can be directed to the corresponding author/s.

## Author Contributions

XG and GW contributed conception and design of the study. SZ and YL searched the literature and singled out included studies. MC performed meta-analysis and wrote the first draft of the manuscript. All authors contributed to manuscript revision, read, and approved the submitted version.

## Conflict of Interest

The authors declare that the research was conducted in the absence of any commercial or financial relationships that could be construed as a potential conflict of interest.

## Abbreviations

95% CI: 95% confidence interval
AD: Alzheimer's disease
BDNF: brain-derived neurotrophic factor
CASI: Cognitive Abilities Screening Instrument
DSM-IV: Diagnostic and Statistical Manual of Mental Disorders (Fourth Edition)
HR: hazard ratio
IL-6: interleukin-6
MeSH: Medical Subject Heading
MOOSE: meta-analysis of observational studies in epidemiology
NINCDS-ADRDA: National Institute of Neurological Diseases and Stroke and the Alzheimer's Disease and Related Disorders Association
NOS: Newcastle-Ottawa Scale
Vit D: vitamin D.
