# Supplementary material for: Grip Strength and the Risk of Cognitive Decline and Dementia: A Systematic Review and Meta-Analysis of Longitudinal Cohort Studies
Source: Front Aging Neurosci. 2021 Feb 4;13:625551. doi: 10.3389/fnagi.2021.625551 (PMC7890203; doi:10.3389/fnagi.2021.625551)
Supplement: Supplementary file 1 [file Table_1.DOCX]

**Supplementary Material 1**

**Specific search strategies for each database**

Pubmed 2020-03-23

Search (((((Strength,muscle[Title/Abstract] OR hand strengths[Title/Abstract] OR strength,hand[Title/Abstract] OR strengths,hand[Title/Abstract] OR grip[Title/Abstract] OR grips[Title/Abstract] OR grasp[Title/Abstract] OR grasps[Title/Abstract])) OR "Hand Strength"[Mesh]) OR "Muscle Strength"[Mesh])) AND (((cognitive impairment[Title/Abstract] OR dementia[Title/Abstract] OR cognitive disorder[Title/Abstract] OR Cognitive Dysfunctions[Title/Abstract] OR Dysfunction, Cognitive[Title/Abstract] OR Dysfunctions, Cognitive[Title/Abstract] OR Cognitive Impairments[Title/Abstract] OR Cognitive Impairment[Title/Abstract] OR Impairment, Cognitive[Title/Abstract] OR Impairments, Cognitive[Title/Abstract] OR Mild Cognitive Impairment[Title/Abstract] OR Cognitive Impairment, Mild[Title/Abstract] OR Cognitive Impairments, Mild[Title/Abstract] OR Impairment, Mild Cognitive[Title/Abstract] OR Impairments, Mild Cognitive[Title/Abstract] OR Mild Cognitive Impairments[Title/Abstract] OR Mild Neurocognitive Disorder[Title/Abstract] OR Disorder, Mild Neurocognitive[Title/Abstract] OR Disorders, Mild Neurocognitive[Title/Abstract] OR Mild Neurocognitive Disorders[Title/Abstract] OR Neurocognitive Disorder, Mild[Title/Abstract] OR Neurocognitive Disorders, Mild[Title/Abstract] OR Cognitive Decline[Title/Abstract] OR Cognitive Declines[Title/Abstract] OR Decline, Cognitive[Title/Abstract] OR Declines, Cognitive[Title/Abstract] OR Mental Deterioration[Title/Abstract] OR Deterioration, Mental[Title/Abstract] OR Deteriorations, Mental[Title/Abstract] OR Mental Deteriorations[Title/Abstract])) OR "Cognitive Dysfunction"[Mesh]) 1111

Filters: English 1050

2000.01.01-2020.03.23 981

Embase

#1 'cognitive defect'/exp

#2 'cognitive dysfunctions':ab,ti OR 'dysfunction, cognitive':ab,ti OR 'dysfunctions, cognitive':ab,ti OR 'cognitive impairments':ab,ti OR 'cognitive impairment':ab,ti OR 'impairment, cognitive':ab,ti OR 'impairments, cognitive':ab,ti OR 'mild cognitive impairment':ab,ti OR 'cognitive impairment, mild':ab,ti OR 'cognitive impairments, mild':ab,ti OR 'impairment, mild cognitive':ab,ti OR 'impairments, mild cognitive':ab,ti OR 'mild cognitive impairments':ab,ti OR 'mild neurocognitive disorder':ab,ti OR 'disorder, mild neurocognitive':ab,ti OR 'disorders, mild neurocognitive':ab,ti OR 'mild neurocognitive disorders':ab,ti OR 'neurocognitive disorder, mild':ab,ti OR 'neurocognitive disorders, mild':ab,ti OR 'cognitive decline':ab,ti OR 'cognitive declines':ab,ti OR 'decline, cognitive':ab,ti OR 'declines, cognitive':ab,ti OR 'mental deterioration':ab,ti OR 'deterioration, mental':ab,ti OR 'deteriorations, mental':ab,ti OR 'mental deteriorations':ab,ti OR 'dementia':ab,ti OR 'cognitive dysfunction':ab,ti

#3=#1 OR #2

#4 'muscle strength'/exp

#5 'grip strength'/exp

#6'strength,muscle':ab,ti OR 'hand strengths':ab,ti OR 'strength,hand':ab,ti OR 'strengths,hand':ab,ti OR 'grip':ab,ti OR 'grips':ab,ti OR 'grasp':ab,ti OR 'grasps':ab,ti OR 'hand strength':ab,ti

#7=#4 OR #5 OR #6

#8=#3 AND #7

#9=#4 AND #8 AND [english]/lim AND [2000-2020]/py 2185

Cochrane

Review 1

Trial 300

2000-2020 301

#1MeSH descriptor: [Cognitive Dysfunction] explode all trees

#2(Cognitive Dysfunctions):ab,ti,kw OR (Dysfunction, Cognitive):ab,ti,kw OR (Dysfunctions, Cognitive):ab,ti,kw OR (Cognitive Impairments):ab,ti,kw OR (Cognitive Impairment):ab,ti,kw OR (Impairment, Cognitive):ab,ti,kw OR (Impairments, Cognitive):ab,ti,kw OR (Mild Cognitive Impairment):ab,ti,kw OR (Cognitive Impairment, Mild):ab,ti,kw OR (Cognitive Impairments, Mild):ab,ti,kw OR (Impairment, Mild Cognitive):ab,ti,kw OR (Impairments, Mild Cognitive):ab,ti,kw OR (Mild Cognitive Impairments):ab,ti,kw OR (Mild Neurocognitive Disorder):ab,ti,kw OR (Disorder, Mild Neurocognitive):ab,ti,kw OR (Disorders, Mild Neurocognitive):ab,ti,kw OR (Mild Neurocognitive Disorders):ab,ti,kw OR (Neurocognitive Disorder, Mild):ab,ti,kw OR (Neurocognitive Disorders, Mild):ab,ti,kw OR (Cognitive Decline):ab,ti,kw OR (Cognitive Declines):ab,ti,kw OR (Decline, Cognitive):ab,ti,kw OR (Declines, Cognitive):ab,ti,kw OR (Mental Deterioration):ab,ti,kw OR (Deterioration, Mental):ab,ti,kw OR (Deteriorations, Mental):ab,ti,kw OR (Mental Deteriorations):ab,ti,kw OR (dementia):ab,ti,kw

#3=#1 OR #2

#4MeSH descriptor: [Muscle Strength] explode all trees

#5(Strength,muscle):ab,ti,kw OR (hand strengths):ab,ti,kw OR (strength,hand):ab,ti,kw OR (strengths,hand):ab,ti,kw OR (grip):ab,ti,kw OR (grips):ab,ti,kw OR (grasp):ab,ti,kw OR (grasps):ab,ti,kw OR (Hand Strength):ab,ti,kw

#6=#4 OR #5

#7=#3 AND #6

2000.01.01-2020.03.23 301

Web of science

#1 (TS=(Cognitive Dysfunctions OR Dysfunction, Cognitive OR Dysfunctions, Cognitive OR Cognitive Impairments OR Cognitive Impairment OR Impairment, Cognitive OR Impairments, Cognitive OR MildCognitive Impairment OR Cognitive Impairment, Mild OR Cognitive Impairments, Mild OR Impairment, Mild Cognitive OR Impairments, Mild Cognitive OR Mild Cognitive Impairments OR Mild Neurocognitive Disorder OR Disorder, Mild Neurocognitive OR Disorders, Mild Neurocognitive OR Mild Neurocognitive Disorders OR Neurocognitive Disorder, Mild OR Neurocognitive Disorders, Mild OR Cognitive Decline OR Cognitive Declines OR Decline, Cognitive OR Declines, Cognitive OR Mental Deterioration OR Deterioration, Mental OR Deteriorations, Mental OR Mental Deteriorations OR dementia OR Cognitive Dysfunction)) AND Language: (English) Index=SCI-EXPANDED Time span=2000-2020

#2 (TS=(Strength,muscle OR hand strengths OR strength,hand OR strengths,hand OR grip OR grips OR grasp OR grasps OR Hand Strength OR muscle strength)) AND Language: (English)

Index=SCI-EXPANDED Time span=2000-2020

#3=#2 AND #1 1312
