# Supplementary material for: Grip Strength and the Risk of Cognitive Decline and Dementia: A Systematic Review and Meta-Analysis of Longitudinal Cohort Studies
Source: Front Aging Neurosci. 2021 Feb 4;13:625551. doi: 10.3389/fnagi.2021.625551 (PMC7890203; doi:10.3389/fnagi.2021.625551)
Supplement: Supplementary file 2 [file Table_2.DOCX]

**Supplementary Table 1**

**Quality assessment of the included studies**

| **Study** | **Selection** | **Comparability** | **Outcome** | **Total** |
| --- | --- | --- | --- | --- |
| Doi,2019 | 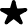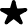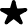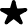 | 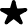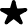 | 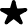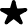 | 8 |
| Jeong,2018 | 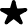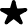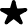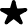 | 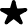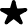 | 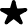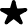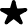 | 9 |
| Sibbett, 2018 | 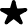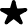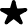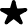 | 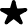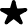 | 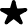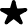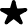 | 9 |
| Heward, 2018 | 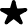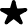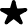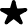 | 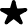 | 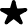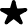 | 7 |
| Sanghoon Jeong,2018 | 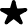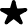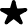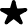 | 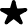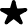 | 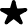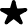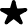 | 9 |
| Hooghiemstra,2017 | 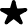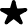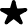 | 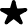 | 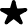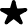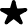 | 7 |
| Camargo,2016 | 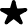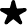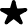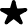 | 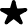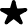 | 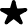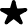 | 8 |
| Veronese,2016 | 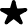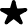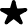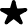 | 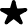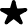 | 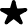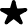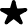 | 9 |
| Gray,2013 | 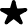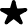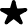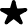 | 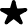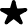 | 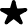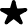 | 8 |
| Sattler,2011 | 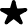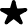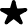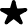 | 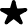 | 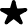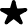 | 7 |
| Boyle,2009 | 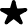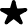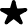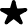 | 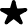 | 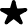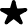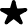 | 8 |
| Buchman,2007 | 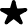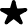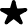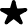 | 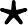 | 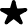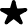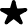 | 8 |
| Kim,2019 | 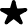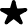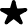 |  |  | 9 |
| Moon,2016 |  |  |  | 7 |
| Hatabe,2020 |  |  |  | 9 |
