# Supplementary material for: Grip Strength and the Risk of Cognitive Decline and Dementia: A Systematic Review and Meta-Analysis of Longitudinal Cohort Studies
Source: Front Aging Neurosci. 2021 Feb 4;13:625551. doi: 10.3389/fnagi.2021.625551 (PMC7890203; doi:10.3389/fnagi.2021.625551)
Supplement: Supplementary file 3 [file Table_3.DOCX]

**Supplementary Figures**

**Meta-analysis, sensitivity analysis and publication bias in this study**


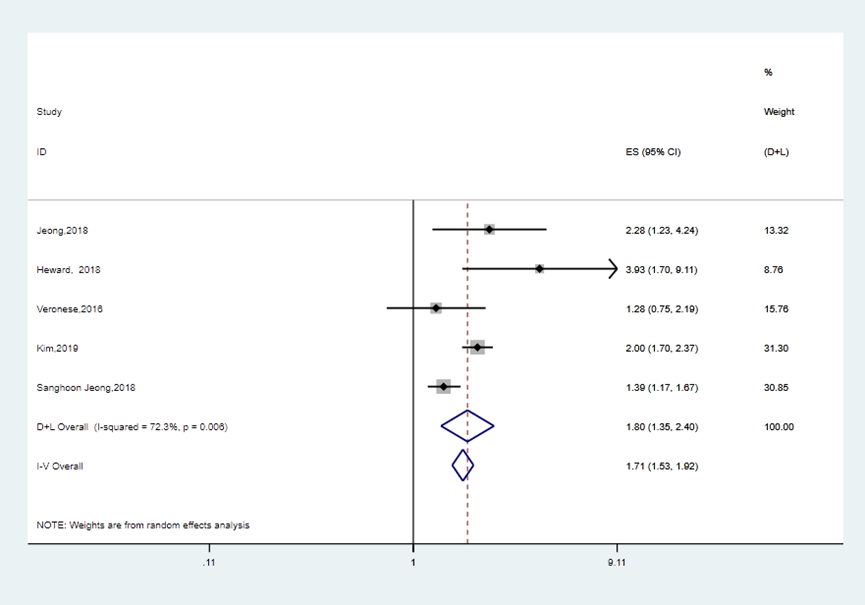


**Figure 1a** **Meta-analysis of the association between grip strength and cognitive decline in all five studies**

Pooled HR of overall five studies on the association between grip strength and cognitive decline.


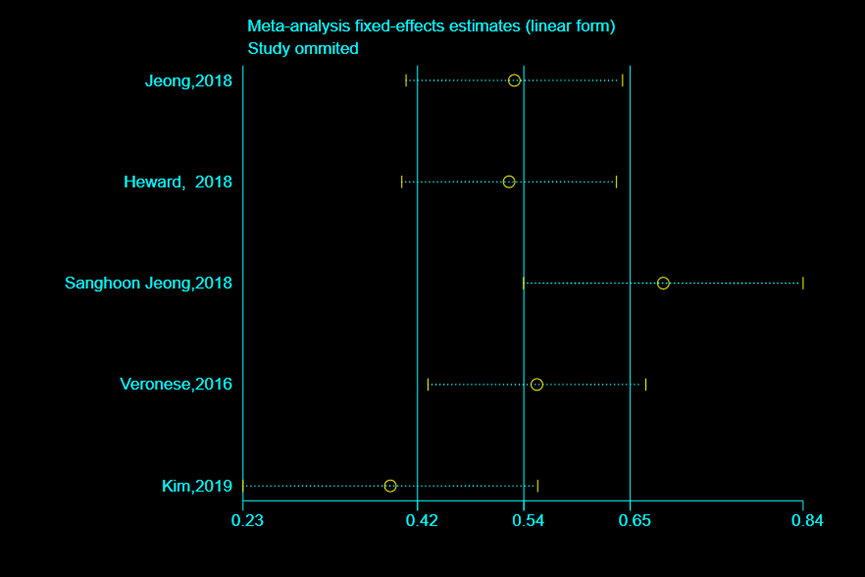


**Figure 1b Sensitivity analysis of meta-analysis of the five studies on the association between grip strength and cognitive decline**

Sensitivity analysis indicated that the study of Sanghoon et al. might be the source of

heterogeneity.


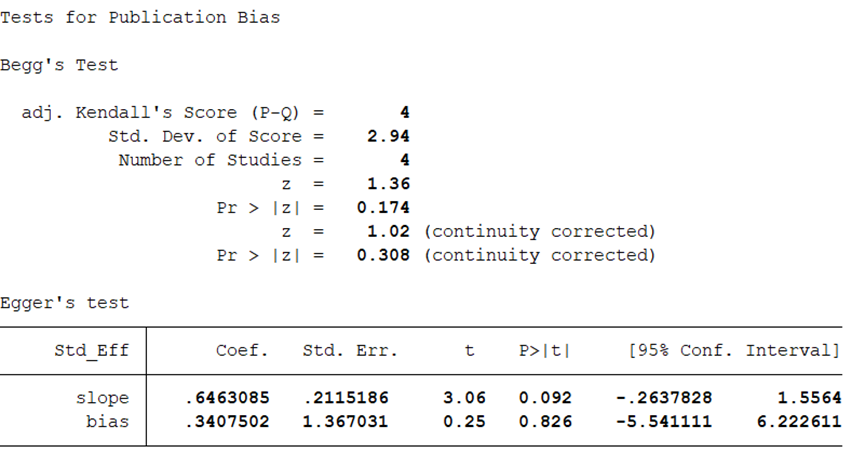
**Figure 1c Publication bias on the association between grip strength and cognitive decline**

There is no publication bias.


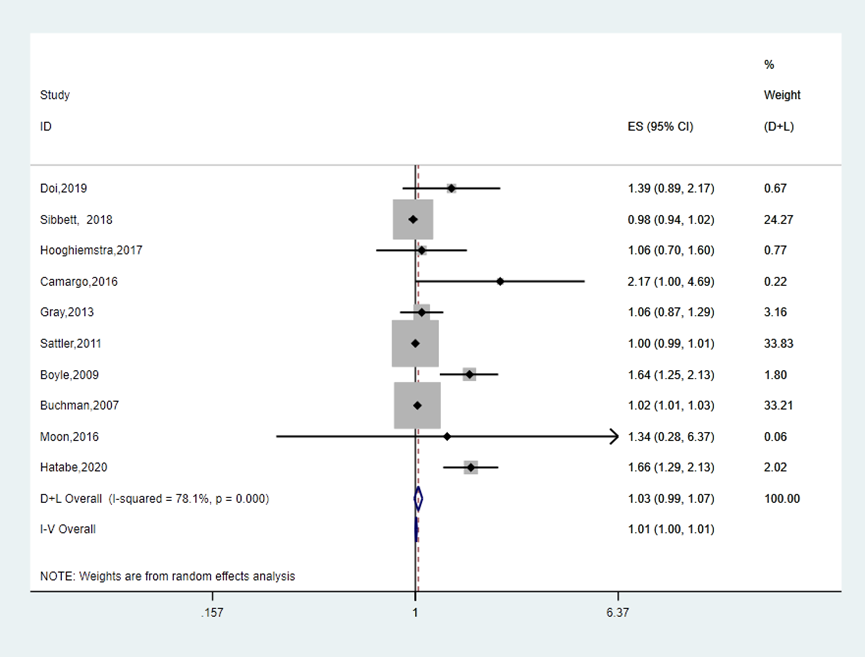


**Figure 2a Meta-analysis of the association between grip strength and dementia in all ten studies**

Pooled HR of overall ten studies on the association between grip strength and dementia.


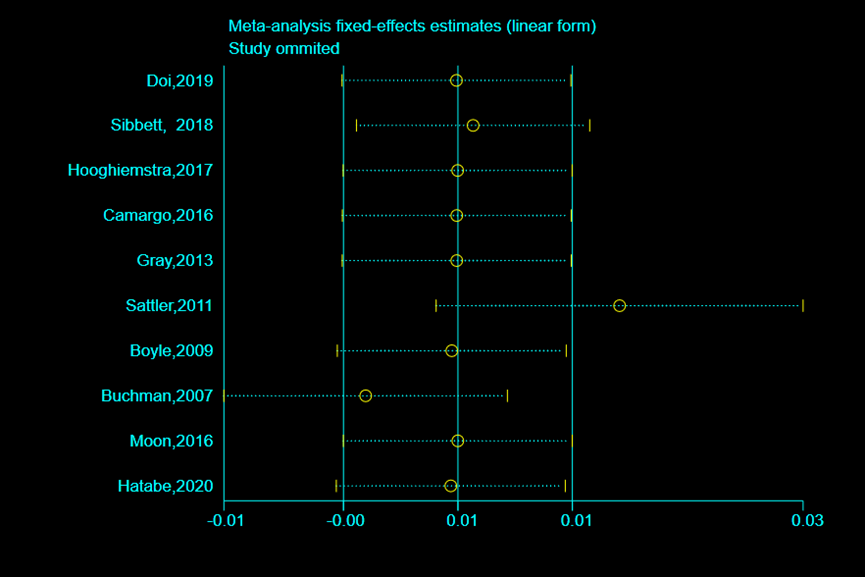


**Figure 2b Sensitivity analysis of meta-analysis of the ten studies on the association between grip strength and dementia**


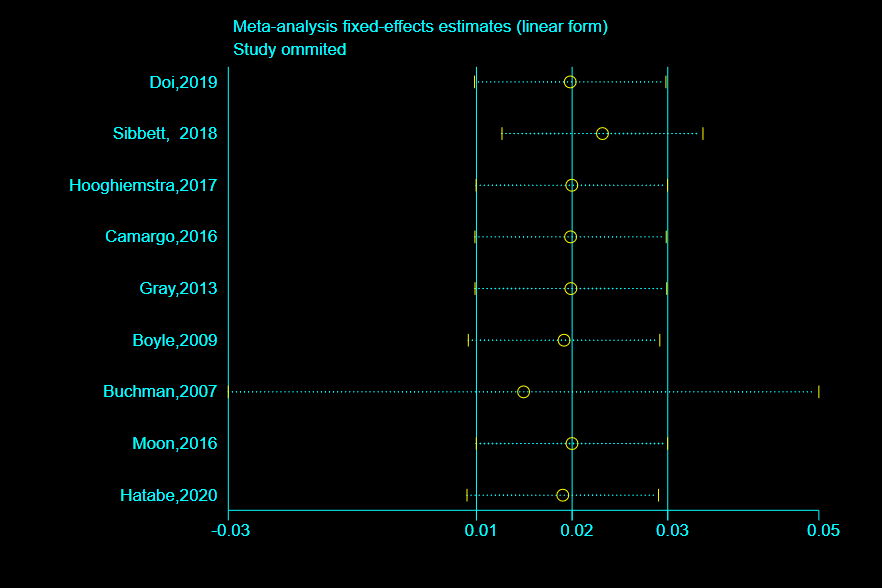


**Figure 2c Sensitivity analysis of meta-analysis of the studies on the association between grip strength and dementia after removing one study**

**
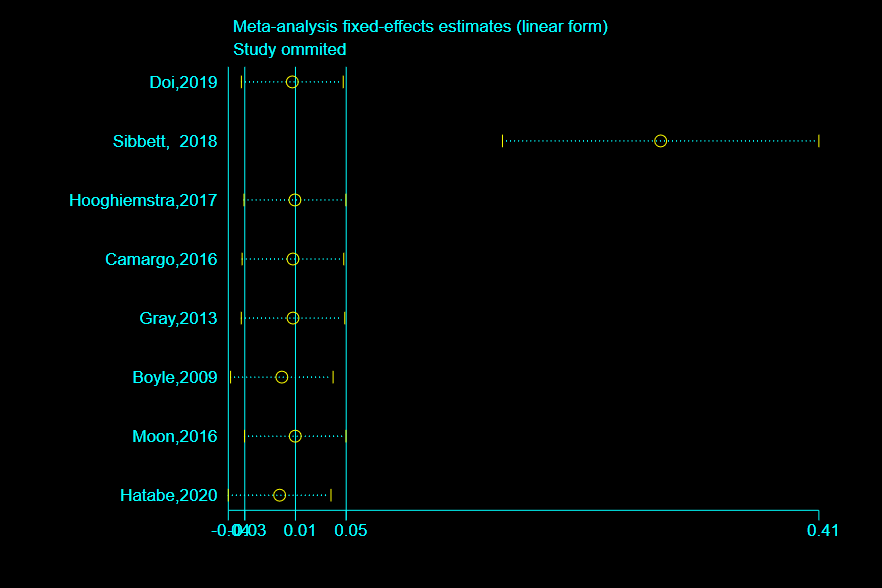
**

**Figure 2d Sensitivity analysis of meta-analysis of the studies on the association between grip strength and dementia after removing two studies**

**
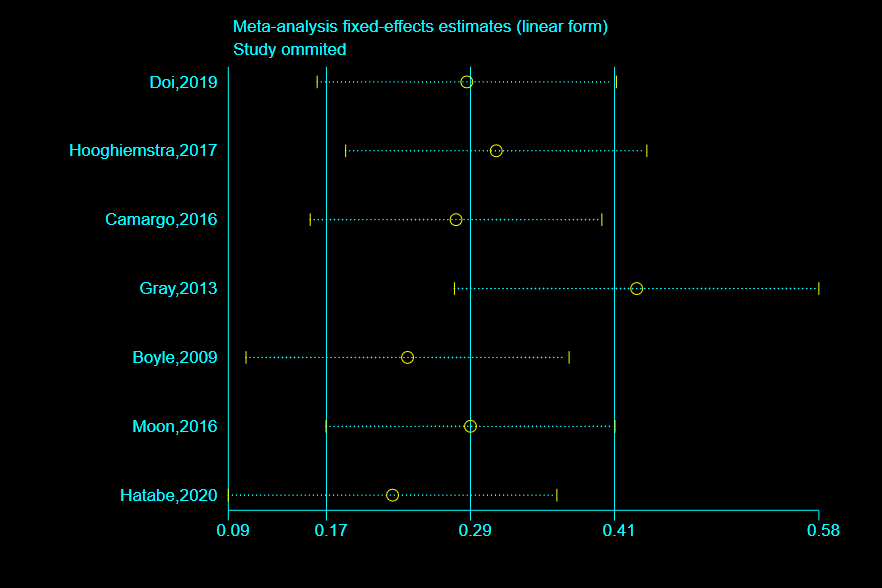
**

**Figure 2e** **Sensitivity analysis of meta-analysis of the studies on the association between grip strength and dementia after removing three studies**

**
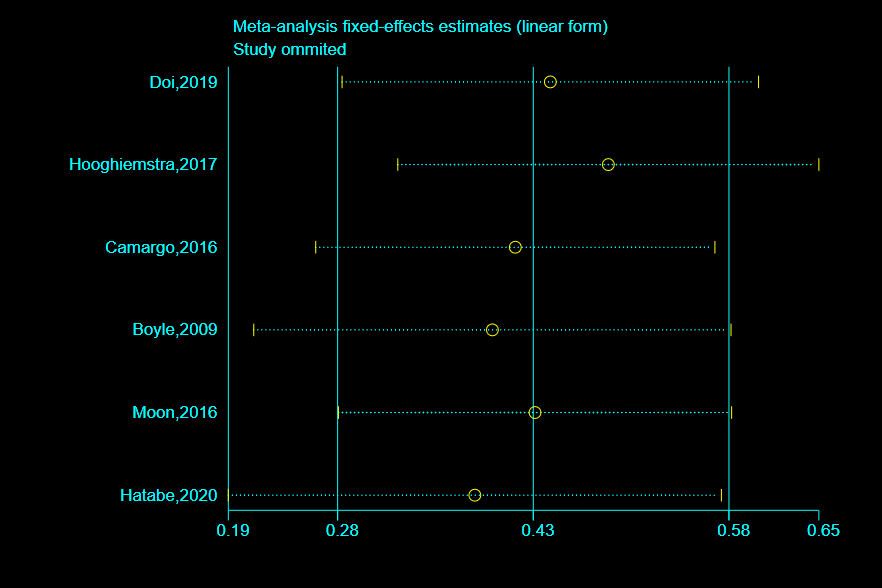
**

**Figure 2f Sensitivity analysis of meta-analysis of the studies on the association between grip strength and dementia after removing four studies**


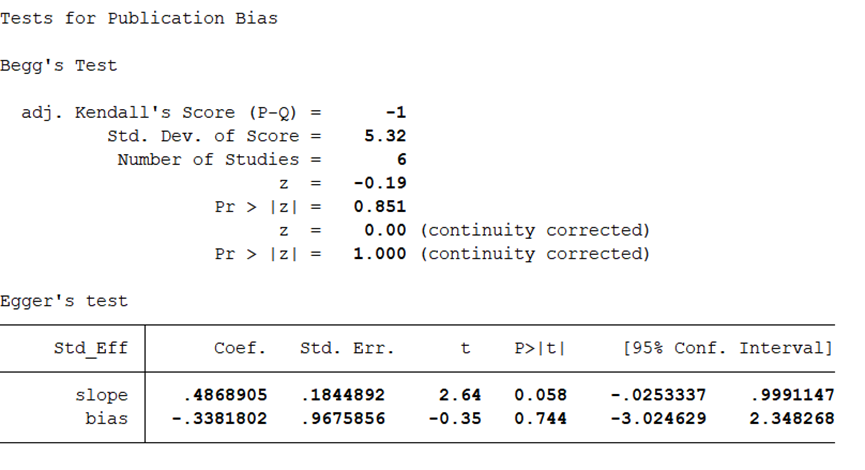
**Figure 2g Publication bias on the association between grip strength and dementia**

There is no publication bias.


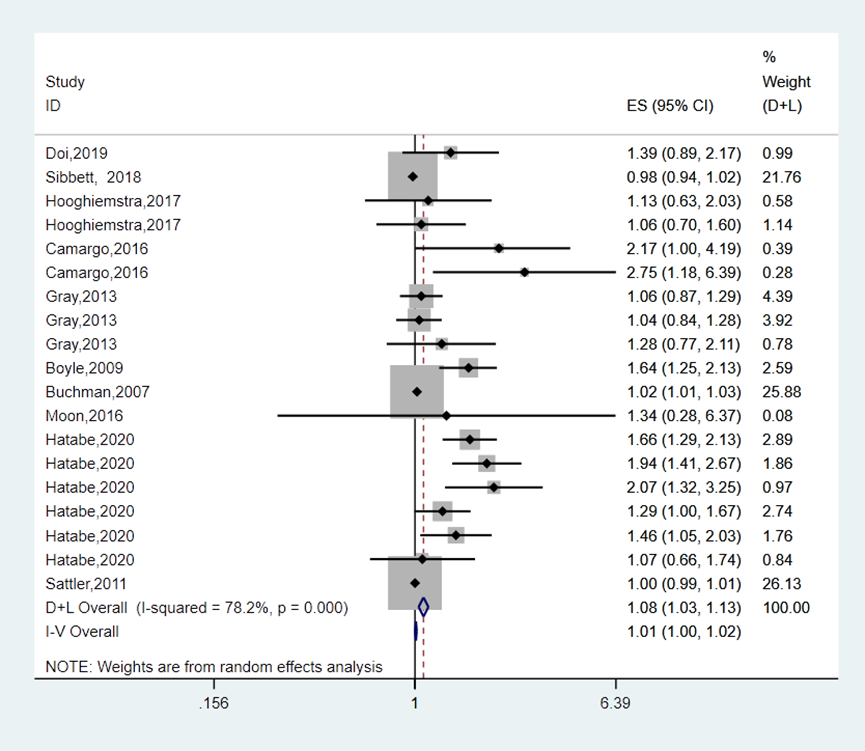


**Figure 3 Meta-analysis of the association between grip strength and all outcomes including dementia, AD and non-AD dementia**

Pooled HR of the association between grip strength and dementia when extracting all outcomes of each study including dementia, AD and non-AD dementia.


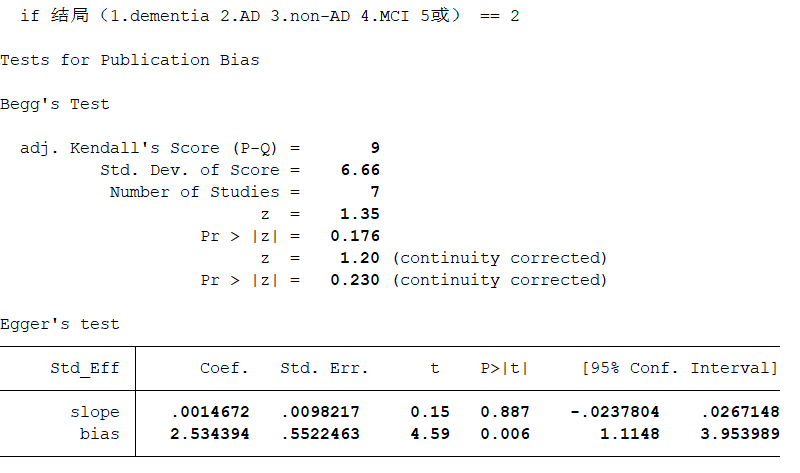


**Figure 4a Publication bias on the association between grip strength and AD**

Begg's test indicated there is no publication bias.

**
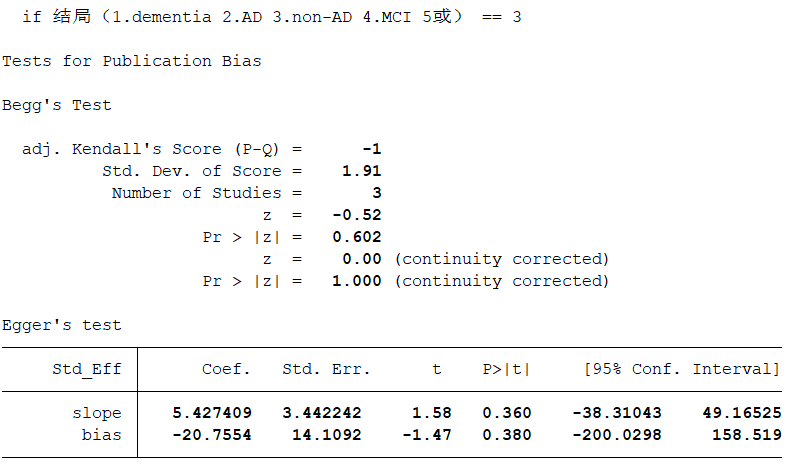
**

**Figure 4b Publication bias on the association between grip strength and non-AD**

There is no publication bias.

**
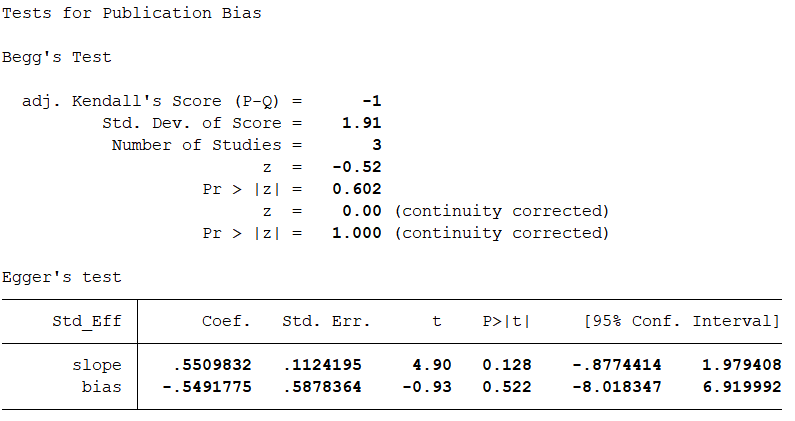
**

**Figure 4c Publication bias on the association between grip strength and dementia if study region is Asia**

There is no publication bias.

**
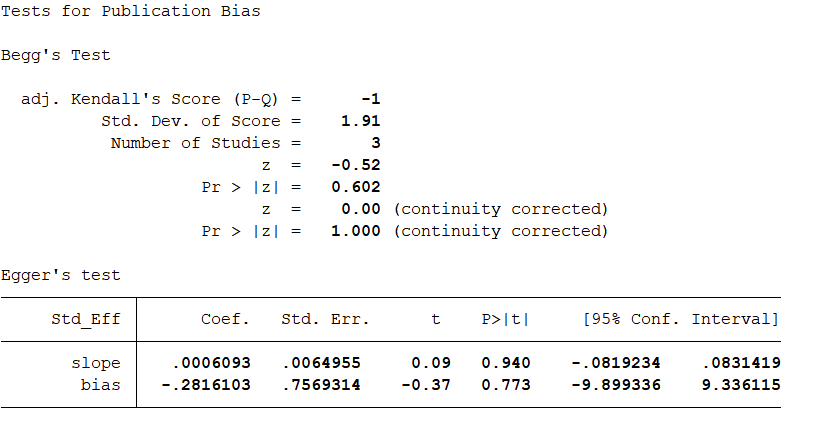
**

**Figure 4d Publication bias on the association between grip strength and dementia if study region is Europe**

There is no publication bias.

**
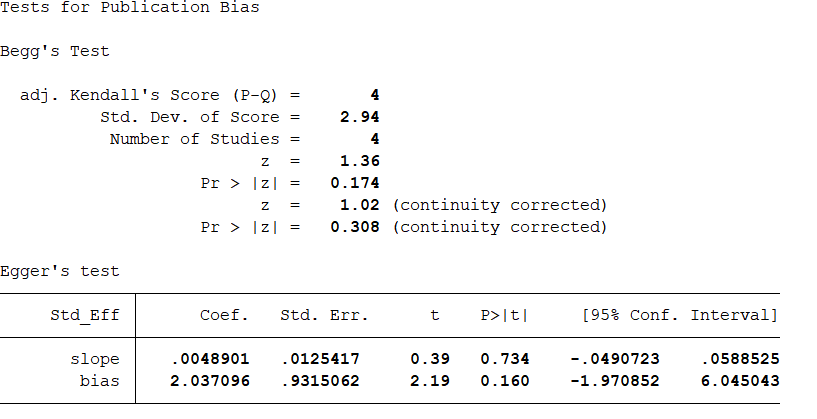
Figure 4e Publication bias on the association between grip strength and dementia if study region is North America**

There is no publication bias.

**
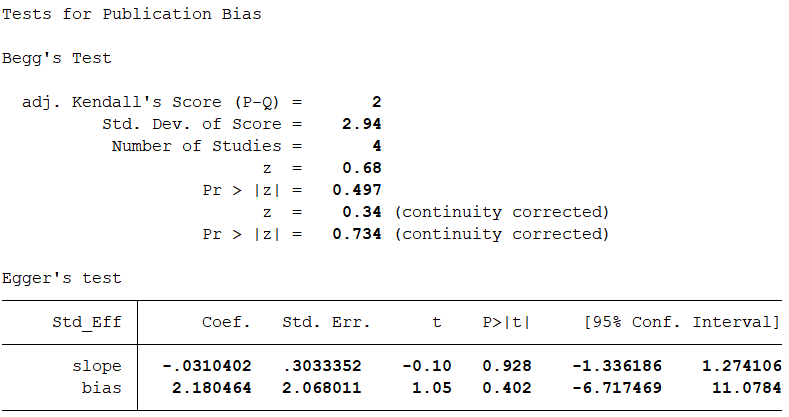
**

**Figure 4f Publication bias on the association between grip strength and dementia if sample size≥1000**

There is no publication bias.

**
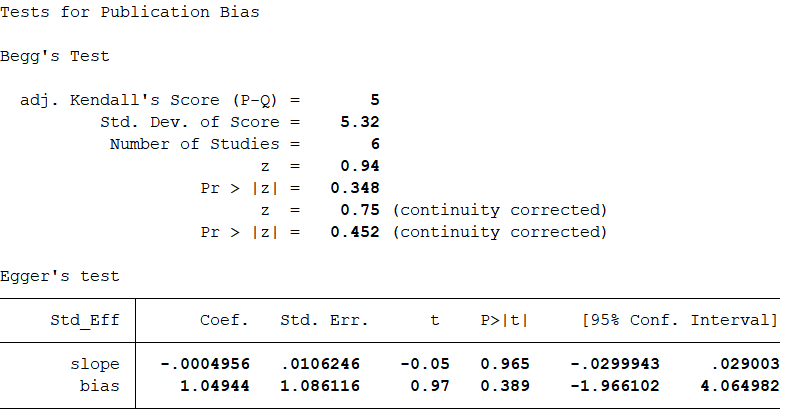
**

**Figure 4g Publication bias on the association between grip strength and dementia if sample size**＜**1000**

There is no publication bias.

**
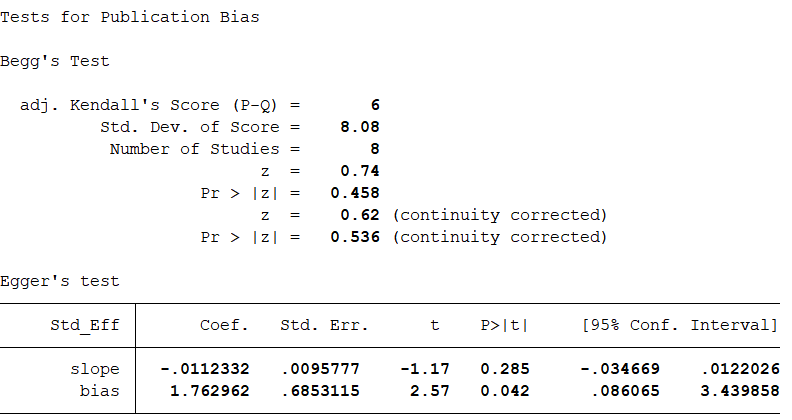
**

**Figure 4h Publication bias on the association between grip strength and dementia if setting is community**

Begg’s test showed there is no publication bias.

**
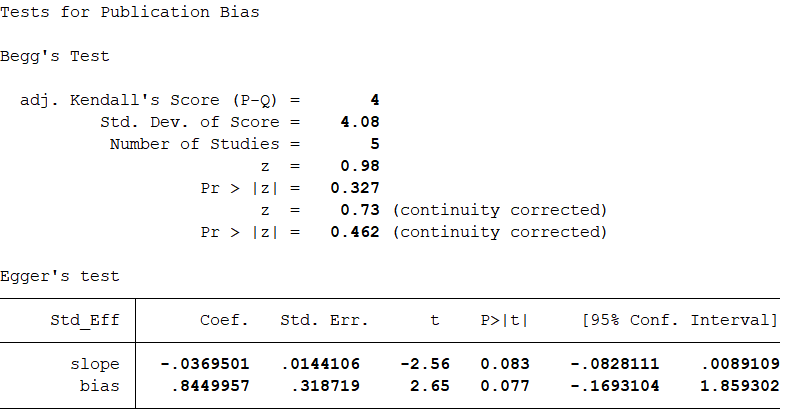
**

**Figure 4i Publication bias on the association between grip strength and dementia if grip strength assessment is dominant hand with handheld dynamometer**

There is no publication bias.

**
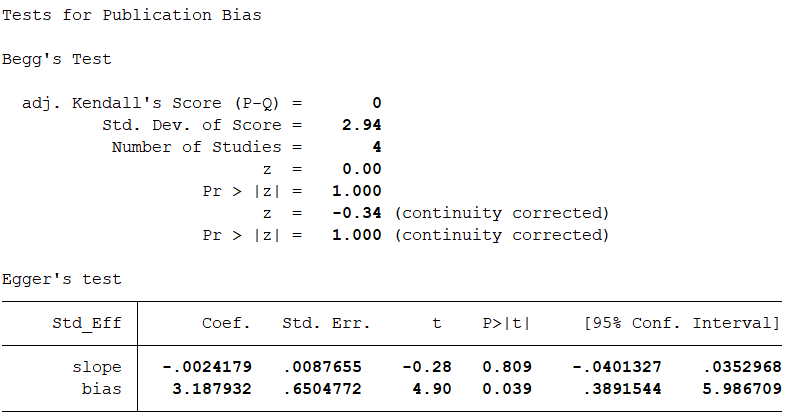
**

**Figure 4j Publication bias on the association between grip strength and dementia if grip strength assessment is both hands with handheld dynamometer**

Begg’s test indicated there is no publication bias.
